# A study on pick cutting properties with full-scale rotary cutting experiments and numerical simulations

**DOI:** 10.1371/journal.pone.0266872

**Published:** 2022-04-14

**Authors:** Qianqian Zhang, Qiuxia Fan, Hongbin Gao, Yulong Wu, Fujing Xu

**Affiliations:** School of Automation and Software Engineering, Shanxi University, Taiyuan, P.R. China; China University of Mining and Technology, CHINA

## Abstract

To investigate the cutting forces on road-header picks, a series of full-scale single-pick rotary cutting tests on sandstone samples were conducted at the National Engineering Laboratory of Coal Mining Machinery and Equipment, China. The primary objective of this study is to optimize the cut spacing and to verify the numerical simulation results. Cutting forces are investigated under different cutting depths and cut spacings. Cut spacing is optimized by analyzing the specific energy, coarseness index, and cutting force. The rock cutting process is simulated on a pick model using the PFC^3D^ software. Rock samples are used as models, and particle assemblies and micro-properties are calibrated by uniaxial compressive strength tests and Brazilian disc tensile strength tests. The optimum ratio of cut spacing to cutting depth for the analyzed sandstone is determined to be in the range of 3 to 4. The experimental results show that a higher coarseness index corresponds to an increased block ratio, and specific energy decreases under optimum cutting conditions. Forces acting on the pick model are determined by simulation. A reasonable agreement exists between the experimental and numerical simulation results regarding the pick forces. The influence of the cut spacing on the rock-breaking effect observed in the experiments is confirmed by numerical simulations. Therefore, numerical simulations using the PFC^3D^ software represent a reliable method for predicting the pick forces.

## Introduction

**Fig 1:**
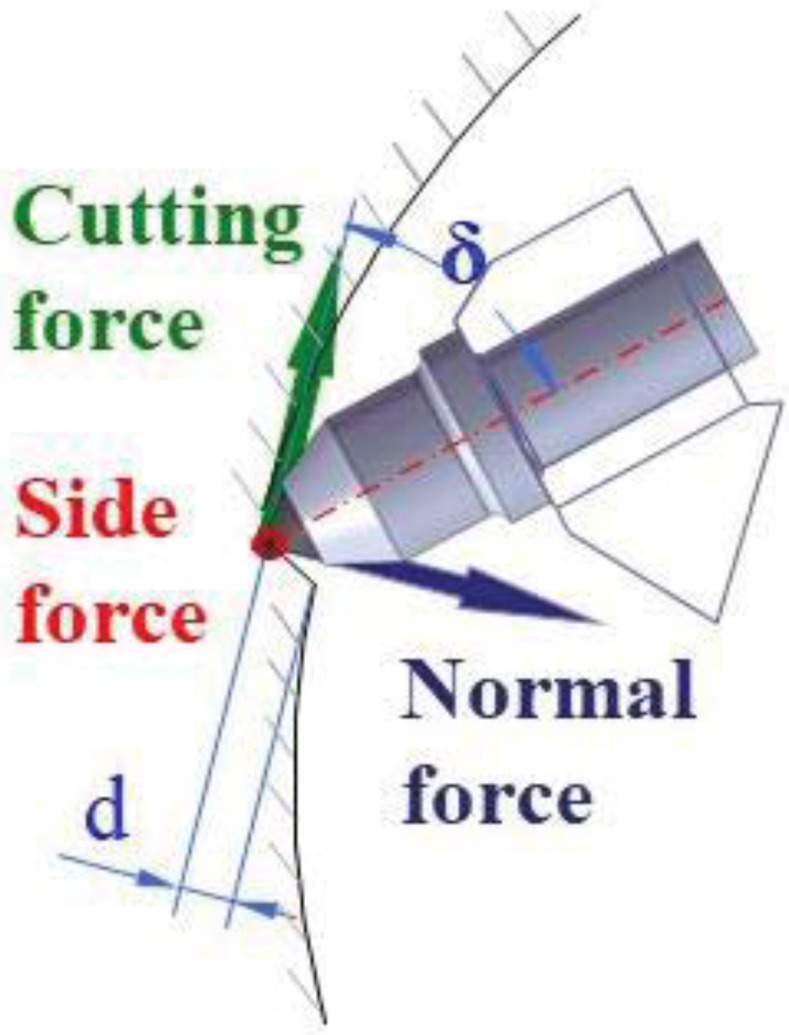
Three orthogonal forces acting on a pick.

**Fig 2:**
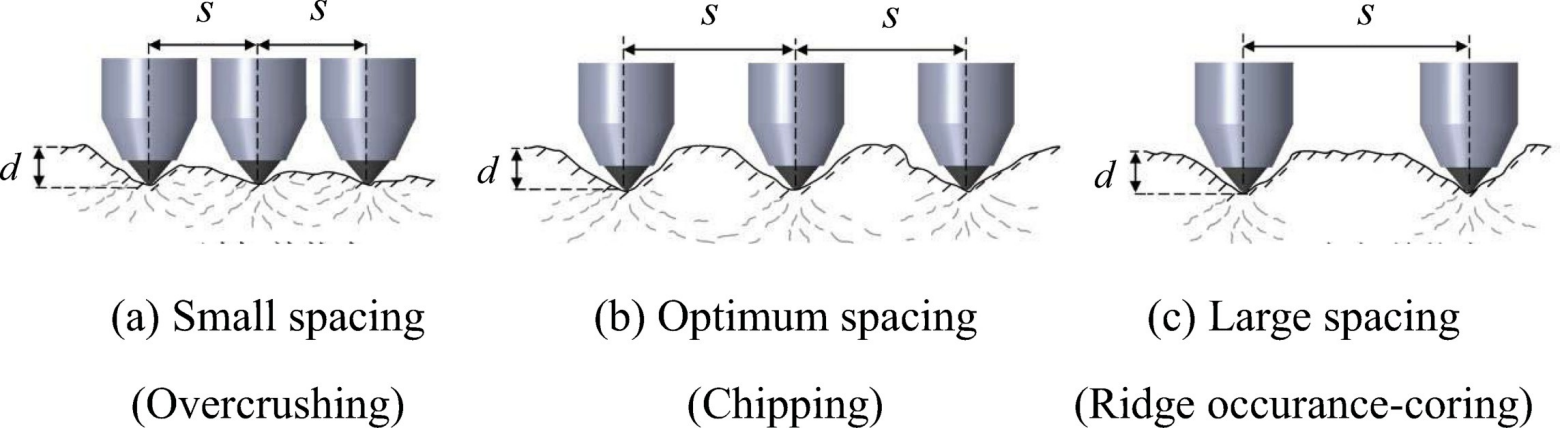
Pick rock cutting patterns based on the cut spacing.

The rock roadway excavation using a road header that generally employs picks as cutting tools has been commonly used in underground coal mining. Therefore, estimating the pick forces, including the normal, cutting, and side forces, acting on a pick is fundamental for determining operational parameters, as shown in Fig 1 [1]. Normal forces are perpendicular to the cutting direction, cutting forces are located along the cutting direction, and side forces are transverse to the cutting direction [2]. Picks on a cutter head have a specific spacing, which is known as cut spacing. The cut spacing is the key parameter for determining the pick arrangement and the number of picks on a cutter head [3]. As shown in Fig 2, cut spacing significantly influences the rock cutting efficiency,. If the cut spacing is too narrow, cutting will be inefficient because the rock is over-crushed. If the cut spacing is too wide, the cutting will also be inefficient because tensile fractures from adjacent cuts cannot form the chips [4]. Thus, it is crucial to determine an optimum cut spacing for given ground conditions.

Several theoretical models and semi-empirical approaches have been commonly used in rock cutting with pointed attack picks [4–11]. Many experimental studies have been carried out to obtain the cutting performance parameters of conical picks, including the pick force, specific energy, and fragmentation. Bilgin et al. [4] conducted the full-scale linear cutting tests under different cutting depths and cut spacings to study conical pick performance on 22 different rock specimens with the compressive strength values varying from 10 MPa to 170 MPa. The pick force and specific energy have a positive effect on rock properties, especially on uniaxial compressive strength and Brazilian tensile strength. Kang et al. [12] designed a new linear cutting machine and investigated the structural stability of a small-capacity linear cutting machine and the feasibility of a new force-measurement method. Shao et al. [13] developed a special thermocouple configuration to investigate the effect of cutting parameters, such as cutting depth, cut spacing, cutting speed, and pick temperature. Wang et al. [14] conducted linear cutting tests using a conical pick under different cutting depths and cut spacings. The results indicated that the cutting depth and cut spacing had a significant effect on the pick force, the ratio of normal force to the cutting force, and specific energy. Wang et al. [15–17] proposed a variety of innovative fault diagnosis methods for faulty gears, that can also be used in the pick wear vibration tests. Dai et al. [18] proposed a new method for recognizing rock failure modes based on the cutting force evolution. A series of tests were performed to validate the method and investigate the critical depth under different test conditions. Comakli et al. [19] developed a new portable linear rock cutting machine. The authors verified its application for selection, design, and proper prediction performance of TBMs instead of a full-scale rock cutting machine.

Numerical simulations have been widely used in pick force prediction. The first trial of a rock cutting numerical simulation using the PFC^3D^ software was performed by Su and Akcin [20]. To validate the feasibility of the PFC^3D^ software in simulated rock cutting tests, a graded particle assembly consisting of four areas shaped like half-cylinders was tested. The numerical results were compared with previously obtained experimental and theoretical results [21]. Moon and Oh [22] proposed a multi-indentation model using the DEM to investigate optimum rock cutting conditions. Van Wyk et al. [23] analyzed tribological interactions to establish a rock-tool model and performed numerical simulations using different tools. The results showed that the cutting depth and pick wear had a significant effect on pick forces during the cutting process. Although numerous attempts have been made to simulate the rock cutting process using the PFC^3D^ software, limited research has been done on the pick cutting process with various cut spacings and cutting depths.

To address the aforementioned shortcoming of the existing literature, a series of full-scale pick cutting tests under different cutting depths and cut spacings were performed in this paper. In addition, the pick cutting process is modeled by the PFC^3D^ software using different cut spacings. The results are compared with the results of the full-scale pick-cutting tests.

## Experimental results

### Full-scale single-pick rotary cutting test

**Fig 3:**
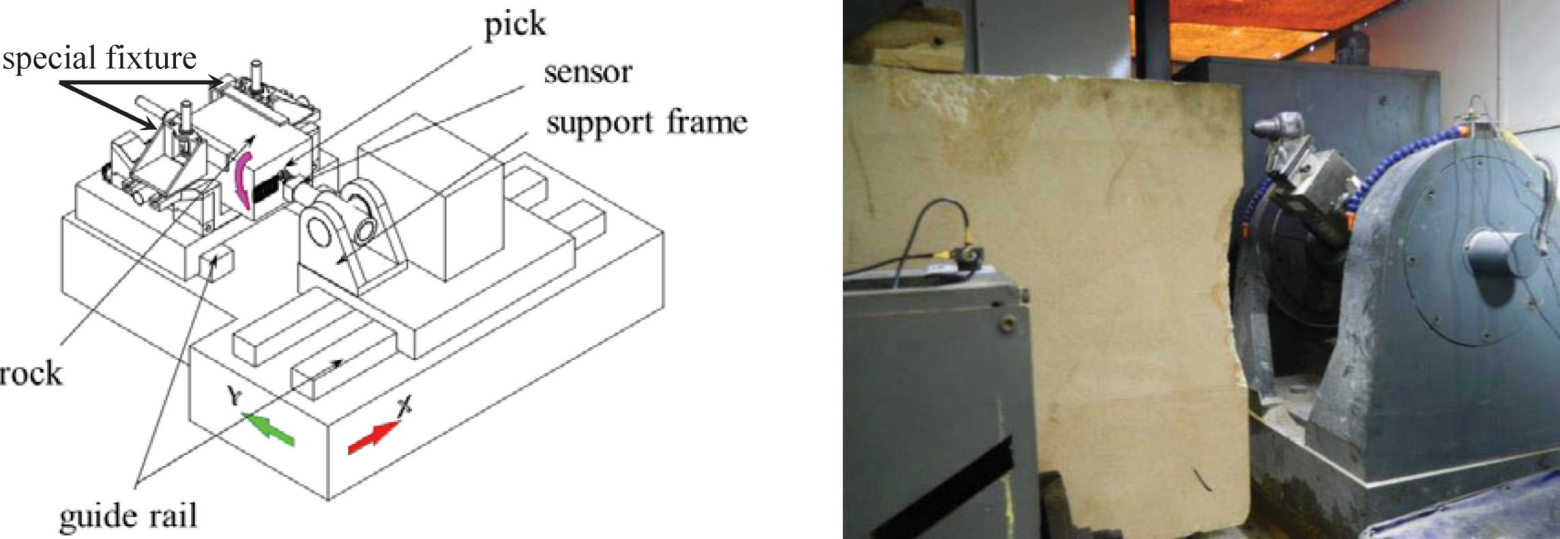
Motion diagrammatic sketch of the rotary cutting machine.

**Fig 4:**
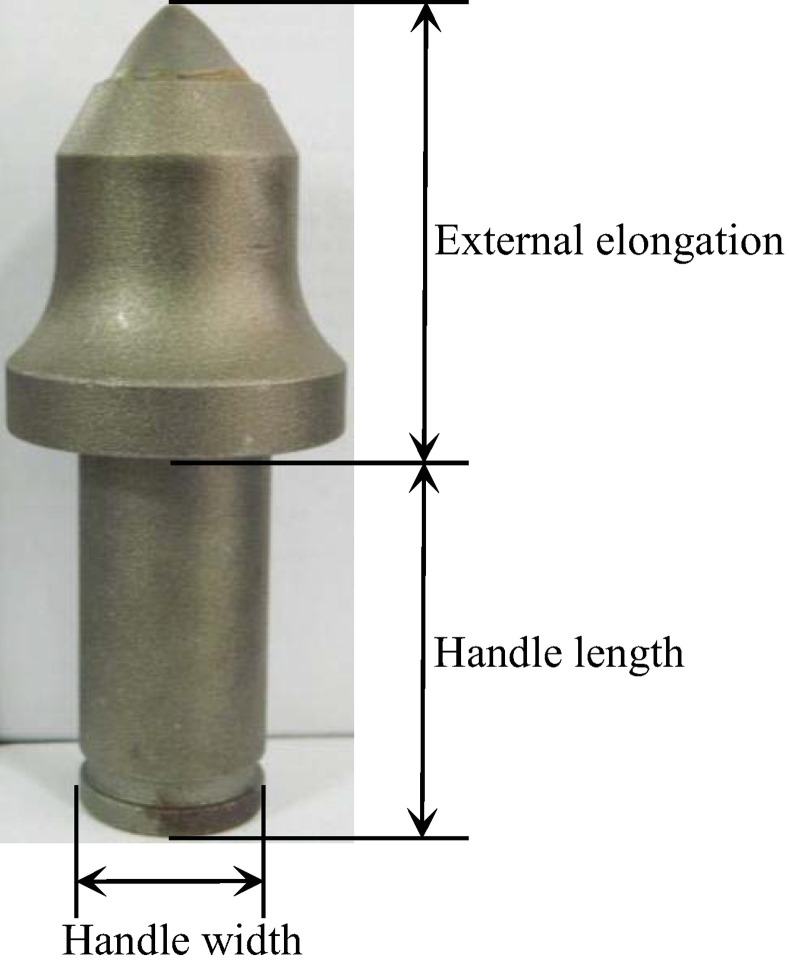
Pick structure.

The full-scale single-pick rotary cutting test was performed using a full-scale rotary cutting machine. This machine, is currently one of the most advanced single-pick rotary cutting machines, at the National Engineering Laboratory of Coal Mining Machinery and Equipment in China. As shown in Fig 3, the rotary cutting machine includs the test platform, control system, test system, and data analysis software,. The cutting machine can continuously monitor the cutting load, vibration, temperature, and dust amount during the tests. The largest rock sample that the cutting machine can accommodate is 1400 mm × 800 mm × 600 mm. The rock was clamped with a special fixture to prevent its movement during the test. A data acquisition system was used to record the pick forces during the cutting process, and the sampling frequency could be adjusted up to 20 kHz. A conical pick manufactured by Sandvik (P5MS-3880-1762) was used for the test. The tip diameter was 25 mm, the pick angle was 80°, the external elongation of the pick was 80 mm, the handle width was 34 mm, and the handle length was 78 mm. The aforementioned is shown in Fig 4.

### Rock mechanical properties test

**Fig 5:**
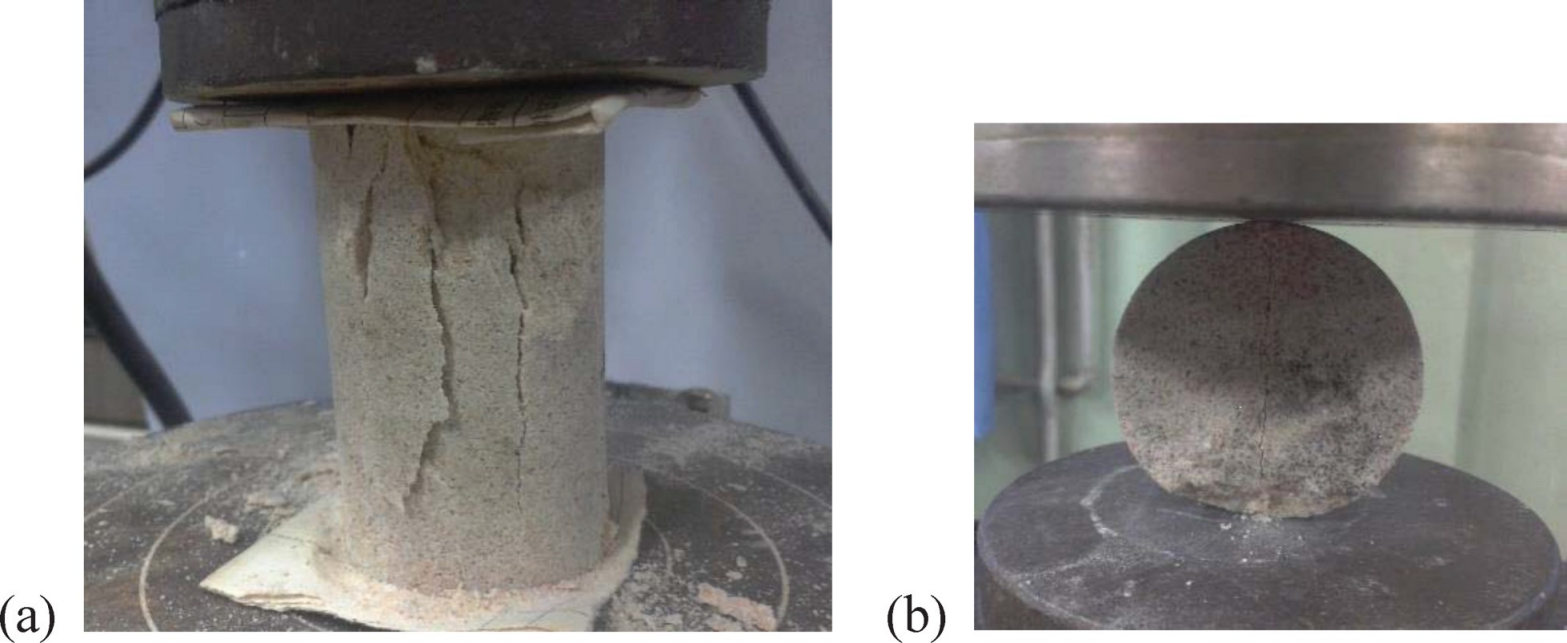
(a) Compressive strength test and (b) Tensile strength test.

Natural sandstone was chosen as a cutting object, and it had a size of 1200 mm × 800 mm × 600 mm. Compressive and tensile properties of the rock are obtained under compressive and tensile loading conditions. Four specimens were evenly drilled from the horizontal and vertical directions on the rock block. Then, they were divided into two groups: A and B. Eight specimens were taken from each group for test repetition, and the results were averaged. For the compressive test, a standard specimen and a cylinder with a diameter of 50 mm and a height-diameter ratio of 2 were used. For the tensile test, a standard specimen and a cylinder with a diameter of 50 mm and a height-diameter ratio of 0.5 were used. For cylindrical specimens, compressive strength and tensile strength tests were carried out on the pressure testing machine, as shown in Fig 5. The results indicated that the natural sandstone had a density of 2340 kg/m^3^, compressive strength of 61.7 MPa, tensile strength of 4 MPa, Young’s modulus (*E*) of 21 GPa, and Poisson’s ratio (*v*) of 0.26.

### Experimental results

#### Influence of cutting depth and cut spacing on specific energy and forces

**Fig 6:**
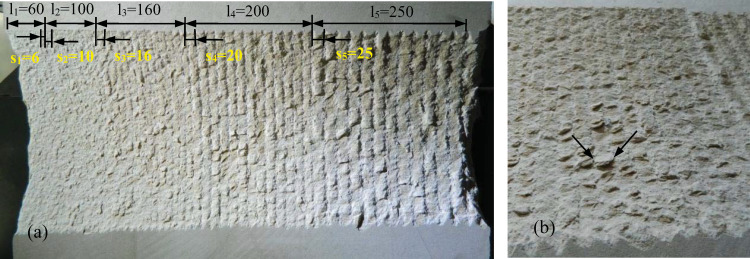
(a) Cut spacing layout, and (b) partial enlargement drawing at s = 16. s_1_~s_5_-Five different cut spacings, mm; l_1_~l_5_-Transverse cutting distance for each cut spacing, mm.

The tests were conducted on the same cutting surface under a constant cutting depth and different cut spacings. The cutting surface of the rock layer at a cutting depth of 6 mm is illustrated in Fig 6A. The cut traces on the rock surface indicate that the rock failure modes produced by different cut spacings varied significantly. When s = 6 mm and s = 10 mm, the repeated cutting phenomenon and an enormous amount of dust were formed during the cutting process. After cutting, a flat surface was formed without obvious cut traces. The rock chips of the two groups were characterized by fine-grained rock fragments, indicating that rock fragments were cut repeatedly during the cutting process. When s = 16 mm, generation of a small number of blocky rock chips has begun, but the thickness was relatively low. The formed rock surface became rough after cutting and cut traces could be observed. Therefore, the grinding cutting process has occurred. When s = 20 mm, a significant number of large fragments were falling from the rock. After cutting, some of the rock chips did not drop onto the lower part of the cutting surface but were semidetached from the rock sample, as shown in Fig 6B. This indicates that the crack formed between two adjacent cuts could be throughout well. Moreover, the aforementioned shows that the cutting spacing of 20 mm was an optimum cutting state at a cutting depth of 6 mm. For s = 25 mm, large rock fragments were falling apart, and ridges were formed on the rough surface. This indicates that the cracks formed between two adjacent cuts could not be throughout each other, and this state was regarded as the unrelieved cutting state.

The test system recorded the components of the pick force during the cutting process. After each test group was completed, the rock chips were collected. The weights of the rock chips were classified according to size, and the trace length of the pick in each test group was calculated.

The specific energy is a representative index that has been used to estimate the cutting efficiency of mechanical cutting tools and is defined by the work required to cut a unit volume of a rock. The specific energy is closely related to the rock properties, rotational speed, cutting power, and tool geometry. The specific energy can be calculated by Eq (1),

$$SE=\frac{{\bar{F}}_{C}L\rho }{m}$$ (1)

where SE is the specific energy (kWh/m^3^), ${\bar{F}}_{C}$ is the mean cutting force of pick (kN), *L* is the cutting distance (mm), *ρ* is the rock density (g/cm^3^), and *m* is the mass of rock chips (g).

**Fig 7:**
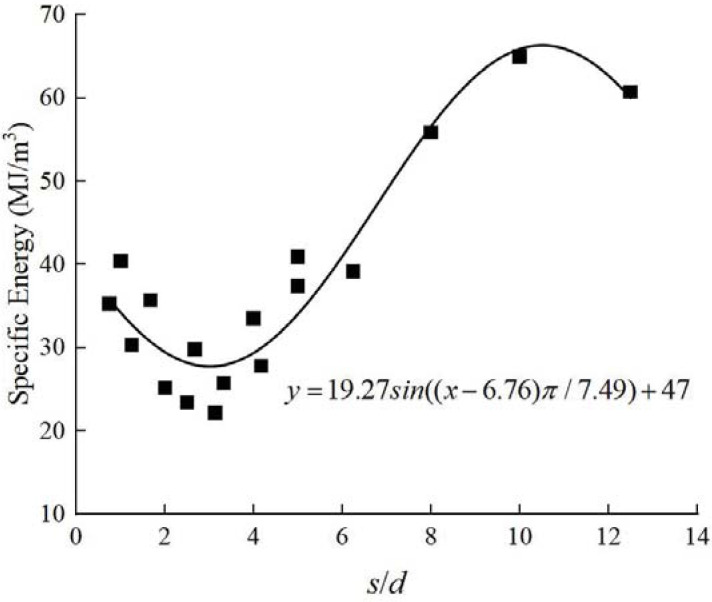
Relationship between SE and the ratio of *s* to *d*.

The SE with different cut spacing and cutting depth is listed in Table 2. When the ratio of cut spacing to cutting depth was less than three, as the cutting depth increased, the specific energy gradually decreased. At the constant cutting depth, the specific energy first decreased and then increased with the cut spacing. To obtain an optimum cutting state, the cut spacing should be reduced when the cutting depth is relatively low. Furthermore, the cut spacing should be increased when the cutting depth is relatively large. The minimum specific energy represents the result of cutting depth and cut spacing combination under different working conditions. The specific energy obtained for different s/d values in the test was recorded and shown in Fig 7. Statistical results indicate that the optimum s/d ratio for this type of sandstone is in the range of 3–4.

**Fig 8:**
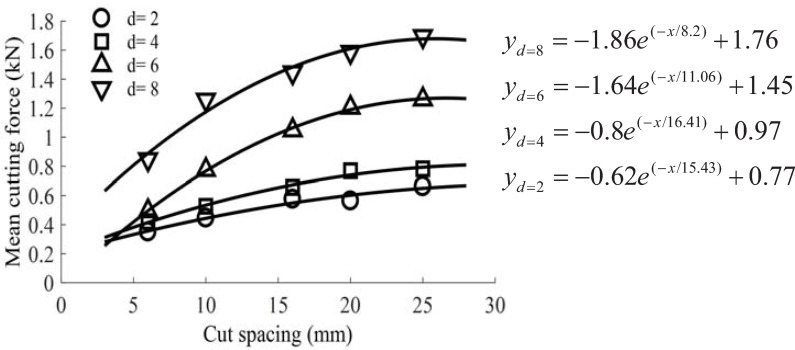
Variation of mean cutting force with cut spacing at different cutting depths.

**Fig 9:**
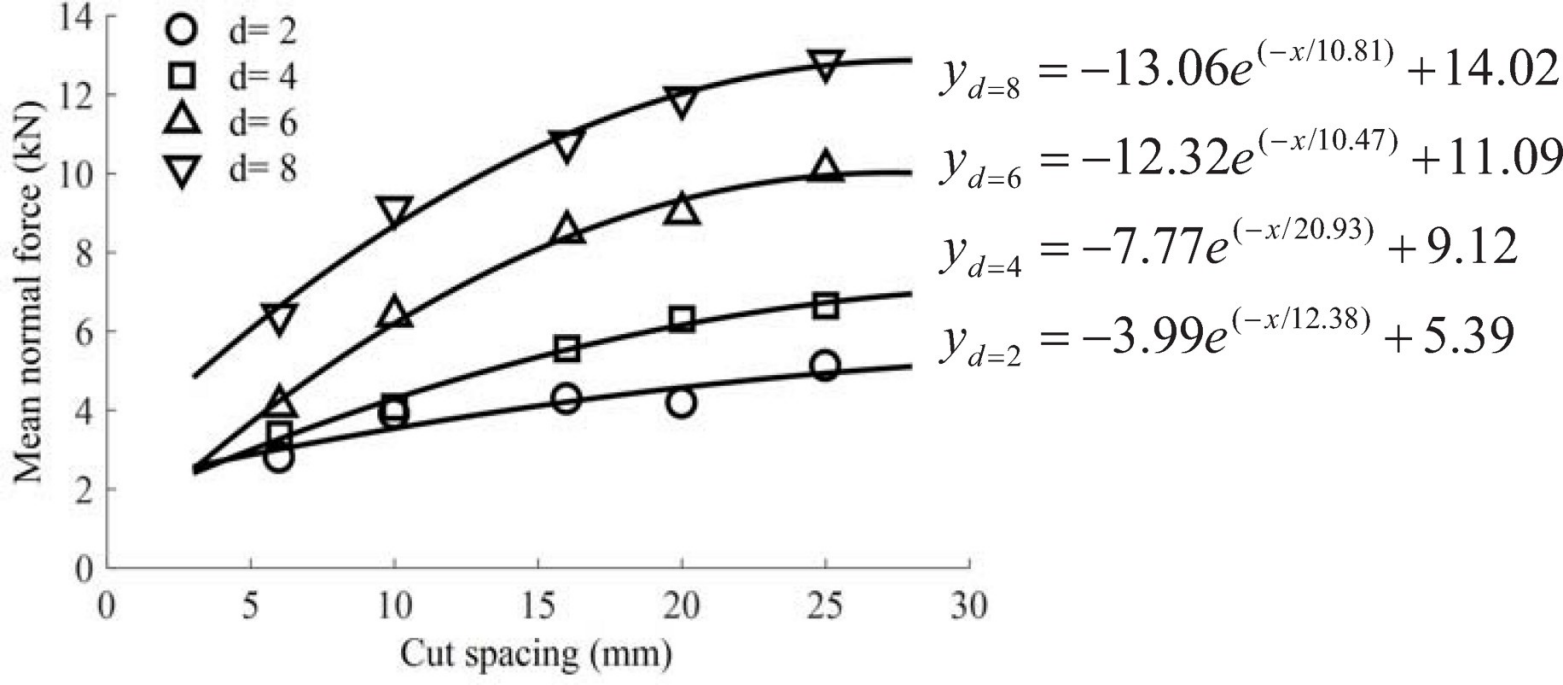
Variation of mean normal force with cut spacing at different cutting depths.

The statistical results also showed that the normal force was the highest, followed by the cutting force, and lastly by the lateral force. The variation curves of average cutting and normal forces with the cut spacing and cutting depth are presented in Figs 8 and 9, respectively. When the cutting depth gradually increased, the average load increased gradually; when the cut spacing increased gradually, the average load acting on the pick fluctuated slightly with small cutting depths of 2 mm-4 mm. When the cutting depth exceeded the value of 4 mm, the average load sharply increased. In addition, when the cut spacing was narrow, the force acting on the pick was lowered due to over-cutting. As the cut spacing increased gradually, the cutting load rapidly increased. If the cracks formed between the adjacent picks were connected, i.e., picks were in the relieved cutting mode, the rock chips would collapse in a block shape. Otherwise, rock ridges would be formed between the two cut traces, i.e., picks would be in the unrelieved cutting mode. As the cut spacing continuously increased, pick force curves reached a maximum value and were relatively smooth.

#### Influence of cutting depth and cut spacing on coarseness index (CI)

To evaluate the cutting efficiency of tunneling equipment, rock chips are commonly analyzed. The coarseness index (CI) is one of the most commonly used evaluation indexes [24]. The CI is a dimensionless quantity defined as a cumulative weight percent of rock chips of varying sizes. The previous research results have indicated the existence of a strong correlation between CI and productivity. More specifically, productivity increases with the CI. A decrease in the specific energy results indicates an increase in the CI and a decrease in the amount of dust [25]. Therefore, the CI is inversely proportional to the specific energy, i.e., a larger CI value corresponds to smaller specific energy.

During the actual rock cutting process (*s* = 10 mm, *d* = 4 mm), the cutting depth started from zero and increased gradually. During the initial stage, rock chips were generated along the front and both sides of the pick. The cutting was dominated by dust, and part of the dust was ejected at high velocity in the opposite direction of the pick movement. By increasing the cutting depth, microcracks formed during the cutting process intersected and continuously expanded, while the number of rock fragments gradually increased. When the cracks formed in the lateral direction intersected with the cracks formed by the adjacent cut, a relatively large block of rock chips was produced. The larger block was strip-shaped and had an average length of 15 mm–20 mm. In the advanced stage of the cutting process, a free surface emerged along the pick movement direction. Lastly, the collapsed rock chips were small to medium-sized and less than 10 mm wide.

**Fig 10:**
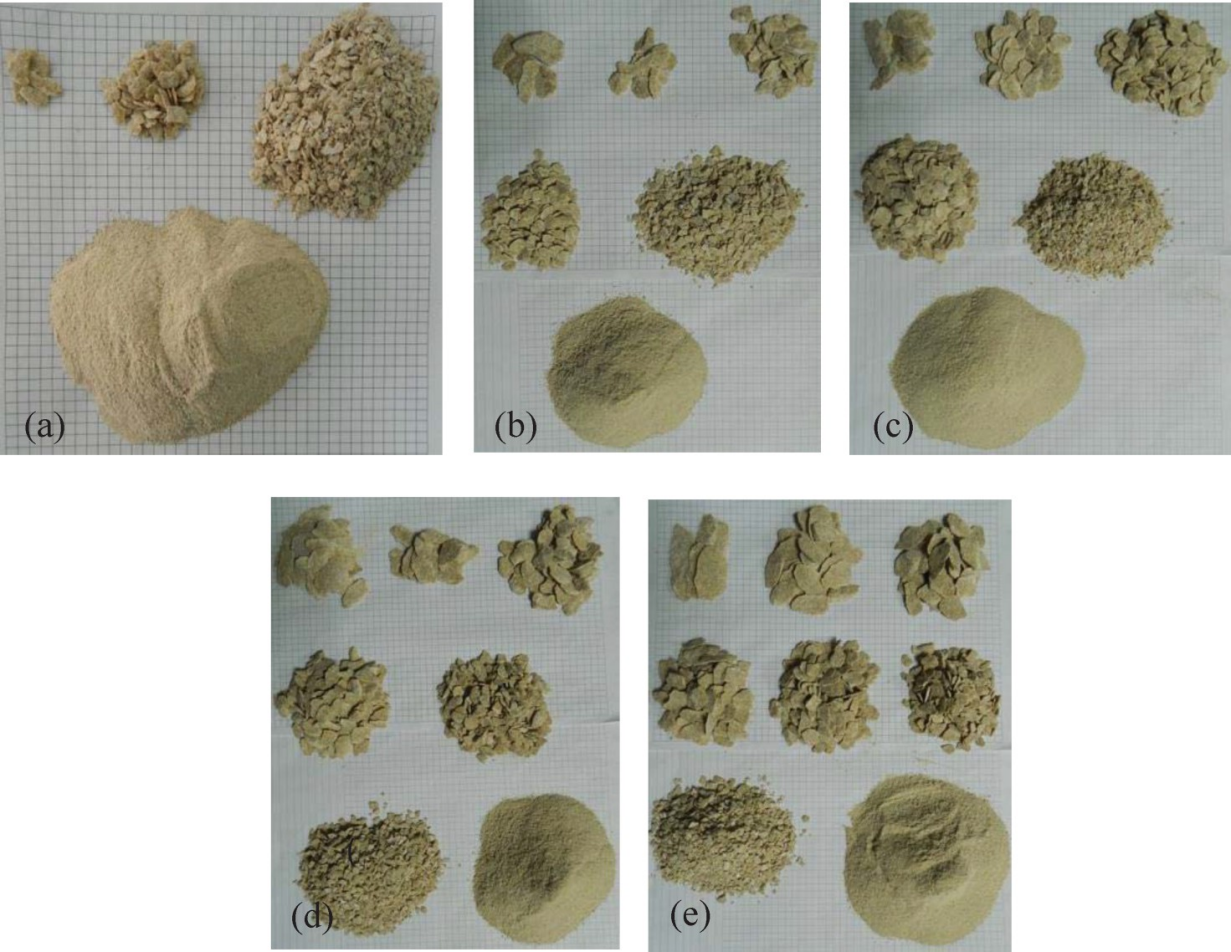
Classification of rock chips for d = 6 mm: (a) s = 6, (b) s = 10, (c) s = 16, (d) s = 20, (e) s = 25 mm.

The rock chips in each group were classified and weighed according to their size, and the corresponding CI values were calculated. The classification diagram of rock chips at the cutting depth of 6 mm and cut spacings of 6 mm, 10 mm, 16 mm, 20 mm, and 25 mm, is presented in Fig 10. It can be observed that the cut spacing has a significant influence on the rock chip size.

The results for the cutting depth of 6 mm, the cut spacing of 25 mm, and the CI of 329.66 are given in Table 1. After a group of tests was completed, the rock chips were classified and weighed according to the size range, and the corresponding weight percentage was calculated. Then, the cumulative weight percentage was determined, and the CI was calculated by summing the cumulative weight percentage.

**Table 1:** Example of CI calculation for *d* = 6 mm and *s* = 25 mm.

Size fraction (mm)	Weight (%)	Cum. Weight (%)
+30	4.13	4.13
-30+25	9.26	13.39
-25+20	8.96	22.35
-20+15	8.69	31.04
-15+10	11.2	42.24
-10+5	8.35	50.59
-5+1.5	15.33	65.92
-1.5	34.08	100
Σ	100	
CI		Σ = 329.66

The CIs with different cutting depths and cut spacings are listed in Table 2. When the cutting depth was relatively small, adjacent picks were in the unrelieved cutting mode, rock chips were primarily in the form of a powder, and the CI was small. When the cut spacing was narrow, the CI value was small and varied slightly with the cutting depth. When the optimum cut spacing to cutting depth ratio was employed, the CI gradually increased with the cutting depth. Therefore, reasonable arrangement of picks on the cutting head and reasonable selection of operating parameters have an important practical significance for rock chip distribution and dust generation.

**Table 2:** Experimental results of forces, SE, and CI in different cutting conditions.

	Cutting depth (*d*/mm)	Cut spacing (*s*/mm)				
		6	10	16	20	25
Mean cutting force (F_C_/kN)	2	0.351	0.447	0.577	0.565	0.663
	4	0.422	0.527	0.656	0.769	0.782
	6	0.494	0.775	1.048	1.204	1.261
	8	0.847	1.257	1.446	1.587	1.696
Mean normal force (F_N_/kN)	2	2.8	3.9	4.28	4.18	5.12
	4	3.39	4.08	5.56	6.31	6.65
	6	4.11	6.4	8.52	9	10.09
	8	6.409	9.129	10.787	11.912	12.843
Specific Energy (SE/kWh/m^3^)	2	67.64	63.19	40.37	35.26	40.9
	4	43.96	35.68	30.31	55.8	33.48
	6	29.75	25.12	64.94	37.39	25.71
	8	23.37	60.69	39.14	27.77	22.16
Coarseness Index (CI)	2	138.06	228.6	189.48	174.64	181.64
	4	161.13	182.28	258.06	226.01	201.49
	6	167.56	185.35	259.93	289.9	353.55
	8	175.63	196.24	299.43	364.58	383.76

**Fig 11:**
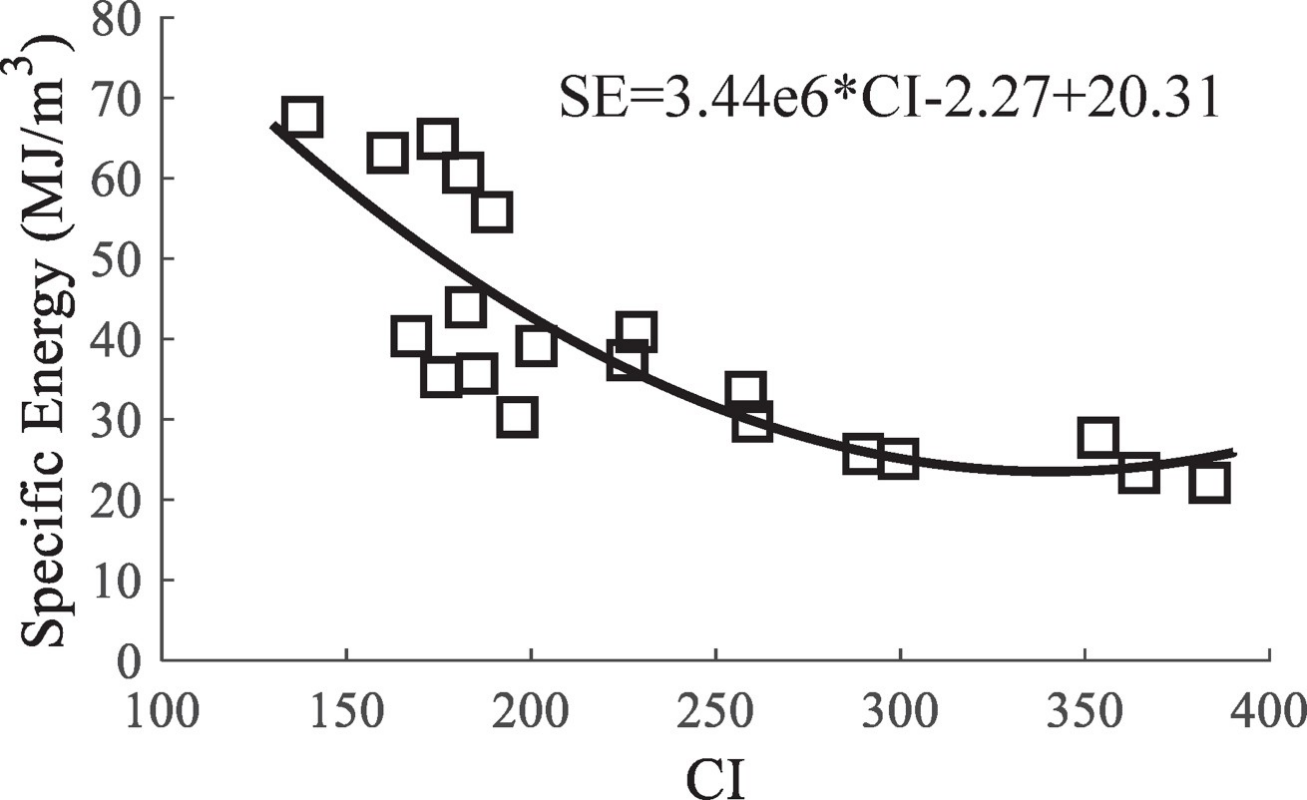
Relationship between specific energy and CI.


Tucdemir et al. [26] defined a relationship between the SE and CI, which is shown in Eq (2). This relationship is appropriate for most rock types and tools. The value of *n* is approximately 1.2 for chisel picks, 1.73.2 for V-type disc cutters, 2.2–4.4 for conical cutters, and 5.5 for CCS disc cutters. Bakar et al. [27] performed the cutting tests on a sandstone under both dry and wet conditions. The results indicated that *n* was 1.04 for the saturated rock and 1.33 for thedry rock when the CCS-type disc cutter was employed. On the other hand, the value of *n* was 4.1 for the saturated rock and 2.3 for the dry rock when the chisel pick was employed. To study the relationship between the SE and CI, a statistical analysis was performed using the exponential fitting, as shown in Fig 11. The analysis results showed a good correlation between the CI and SE; also, CI and SE were inversely proportional.

$$SE=k\;CI^{-n}$$ (2)

where *k* is a function of rock strength and cutting tool parameters, and *n* is related to the type of tools. In this test, the conical pick was used to cut dry rock. The value of *n* obtained from the test result is 2.27, which is consistent with the predicted range according to [28].

## Establishment of numerical simulation model

### PFC^3D^ overview

**Fig 12:**
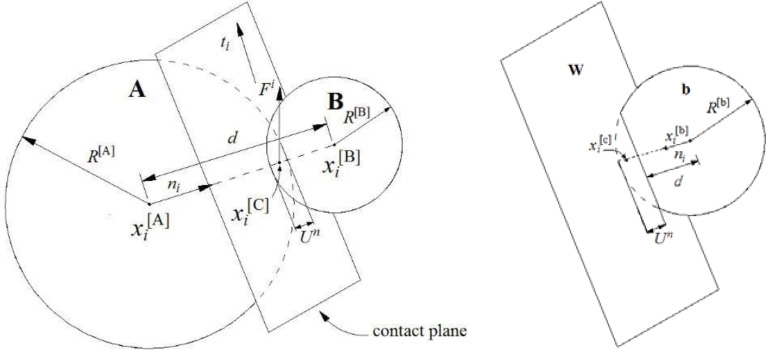
The notation used to describe ball-ball contact and ball-wall contact [30].

Based on the definition of the discrete element method proposed by Cundall and Hart [29], the PFC^3D^ can be regarded as a discrete element code. It allows finite displacements and rotations of discrete bodies, including complete detachment. Moreover, it automatically recognizes new contacts in the calculation progresses. The calculation cycle of the PFC^3D^ is a time-stepping algorithm that repeatedly applies the law of motion to every particle and a force-displacement law to every contact. Moreover, it constantly updates the wall positions. A general view of the ball-ball and ball-wall contacts is shown in Fig 12.

Whenever two particles overlap, a contact is formed at the center of the overlapping region along the straight line connecting the particle centers ($x_{i}^{\left[C\right]}$ in Fig 12), and two linear springs are inserted along with a slider in the shear direction. The force-displacement law is defined for both the ball-ball and the ball-wall contacts. Then, the law is used to calculate unbalanced forces on an individual particle. The motions of particles and walls can be tracked by a pointer. The contact force vector *F*_*i*_ can be decomposed into normal and shear components with respect to the contact plane. Then, the shear component can be computed in an incremental mode. When the contact is formed, $F_{i}^{s}$ is initialized to zero. Every subsequent relative shear-displacement increment $\Delta U_{i}^{s}$ produces an increment in the elastic shear force $\Delta F_{i}^{s}$, which is added to $F_{i}^{s}$. The calculation equations are as follows:

$$F_{i}=F_{i}^{n}n_{i}+F_{i}^{s}t_{i}$$ (3)

$$F_{i}^{n}=K_{}^{n}U_{}^{n}$$ (4)

$$\Delta F_{i}^{s}=-k_{}^{s}\Delta U_{i}^{s}$$ (5)

where *F*_*i*_ is the contact force, $F_{i}^{n}$ and $F_{i}^{s}$ denote the normal and shear force components, *K*^*n*^ is the contact normal stiffness, *U*^*n*^ is the overlap, *n*_*i*_ and *t*_*i*_ are the unit normal and shear vectors, $\Delta F_{i}^{s}$ is the increment of elastic shear force, *k*^*s*^ is the shear stiffness, and $\Delta U_{i}^{s}$ is the relative shear-displacement increment.

Particle motion, including movement and rotation, depends on the force and torque acting on the particle. Motion description is given by Eqs (6) and (7) and is integrated using a centered finite difference procedure involving a timestep of Δ*t*. Parameters ${\dot{x}}_{i}$ and ${\dot{w}}_{i}$ are computed at the mid-intervals of *t* ± *n*Δ*t*/2, while parameters *x*_*i*_, ${\ddot{x}}_{i}$, ${\dot{w}}_{i}$, *F*_*i*_, and *M*_*i*_ are computed at the primary intervals of *t* ± *n*Δ*t*. Vector equation can be written as follows (Itasca Consulting Group, 2006):

$$F_{i}=m({\ddot{\text{x}}}_{i}-g_{i})\;(\text{translational}\;\text{motion})$$ (6)

$$M_{i}=I{\dot{w}}_{i}=(\frac{2}{5}mR_{}^{2}){\dot{w}}_{i}\;(\text{rotational}\;\text{motion})$$ (7)

where *F*_*i*_ is the resultant force, i.e. the sum of all externally applied forces acting on the particle; *m* is the total mass of the particle; and *g*_*i*_ is the body force acceleration vector (e.g., gravity loading). The parameter *M*_*i*_ is the resultant moment acting on the particle, *I* is the moment of inertia, *I* is the radius of the spherical particle, and *w* is the angular velocity.

### Calibration of micro parameters and establishment of rock-tool model

The particle formation program proposed by Potyondy and Cundall was used to construct a cylindrical model with a length to diameter ratio of 2:1 in the PFC^3D^ [31]. To avoid the effects of the size on the calibration results, the model size was the same as that of the test sample. The smaller the particle radius was, the more accurately the particle model would reflect the mechanical rock properties. However, if the number of particles was overly large, the simulation time would be significantly increased. Thus, to ensure that the number of particles contacted by the pick was higher than three during the initial cutting stage, the particle radius was set in the range of 0.6 mm-0.72 mm. The parallel bond contact model was used to build the rock model in the PFC^3D^. Micro parameters of the rock model were calibrated through the simulation tests of compressive strength and tensile strength which are consistent with the experimental conditions. A large number of repeated simulation tests were conducted through constant modification of microscopic parameters. When the microscopic parameters are calibrated, the elastic modulus, Poisson’s ratio, tensile strength and compressive strength are taken as the evaluation indexes, and the corresponding numerical results are directly output without comparing the stress-strain curves between simulation results and experimental results. Finally, a set of microscopic parameters were obtained that can generate a rock particle model with very close physical properties, as shown in Table 3. The macro properties of the rock sample are given in Table 4.

**Table 3:** Micro properties of rock model.

Particle properties					Bond properties				
*ρ*	*R*_max_/*R*_min_	*E*_c_	*k*_n_/*k*_s_	*μ*	$\bar{\lambda }$	${\bar{E}}_{c}$	${\bar{k}}_{n}/{\bar{k}}_{s}$	${\bar{\sigma }}_{c}$	${\bar{\tau }}_{c}$
2.34	1.2	24	1	0.5	1	24	1	77±10	77±10

*ρ* = density (g/ cm^3^); *R*_*max*_ / *R*_*min*_ = radius ratio; *E*_*c*_ = Young’s modulus (GPa); *k*_*n*_ / *k*_*s*_ = ratio of normal to shear stiffness for particle properties; *μ* = friction coefficient; $\bar{\lambda }$ = radius multiplier; ${\bar{E}}_{c}$ = Young’s modulus (GPa); ${\bar{k}}_{n}/{\bar{k}}_{s}$ = ratio of normal to shear stiffness for parallel bond properties; ${\bar{\sigma }}_{c}$ = tensile strength (MPa); ${\bar{\tau }}_{c}$ = shear strength (MPa).

**Table 4:** Macro properties of the rock specimen.

Property	Laboratory test	Simulation
***ρ* (g/ cm** ^**3**^ **)**	2.34	2.34
**Compression strength (MPa)**	61.7	61.5
**Tensile strength (MPa)**	4.1	4.5
***E* (GPa)**	21	19.8
***v***	0.26	0.26

**Fig 13:**
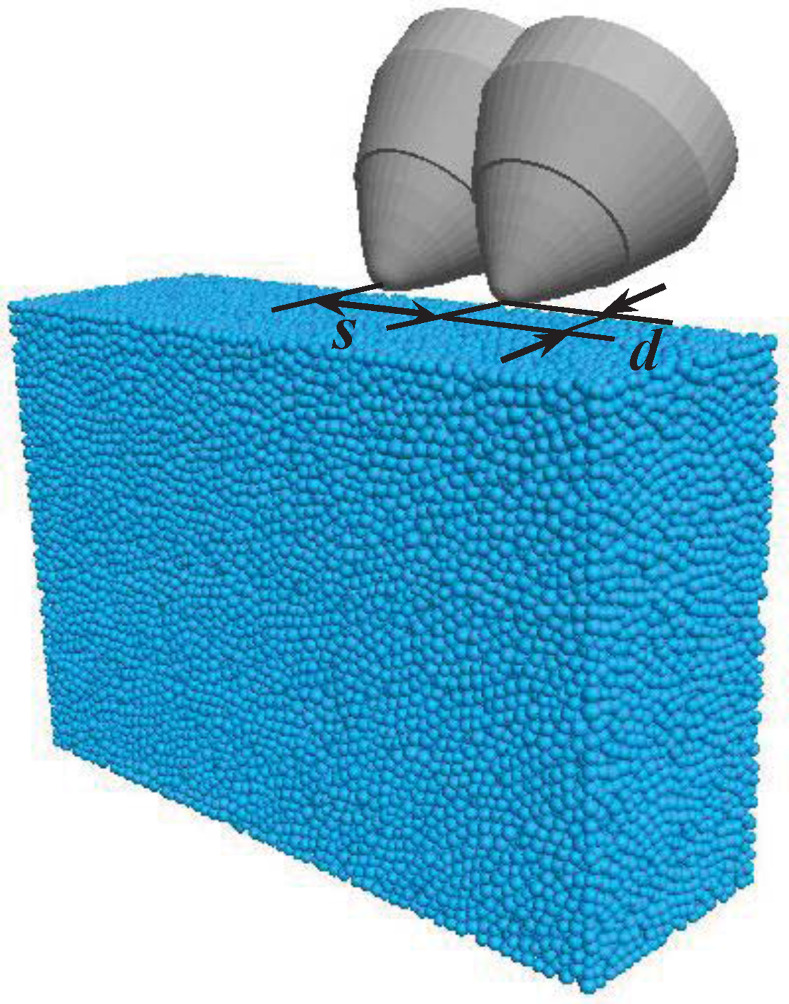
Particle assembly-tool model with different cut spacings.

The pick model was established based on the geometric parameters of Sandvik p5ms-3880-1762. Compressive and tensile strength parameters represent the physical properties of the rock. Based on the calibrated micro parameters, the particle diameter range is set as 0.6–0.72 mm. Micro properties including particle properties and bond properties shown in Table 3, are taken as input parameters to establish the particle assembly model whose size is 40 mm×120mm×80mm, as shown in Fig 13. A simulation program was developed according to the experimental conditions. First, the first pick was utilized to cut the rock model with an angular velocity of 2.72 rad/s, and a cutting distance of 58 mm. Then, the current pick was deleted, and the second pick was imported. The cut spacings were 6 mm, 10 mm, 16 mm, 20 mm, 25 mm, and 30 mm, while the cutting depth was 4 mm. The ratio of the cut spacing and cutting depth was between 1.5 and 7.5. During the simulation cutting process, pick forces acting on the rock model were recorded.

## Results and discussion

**Fig 14:**
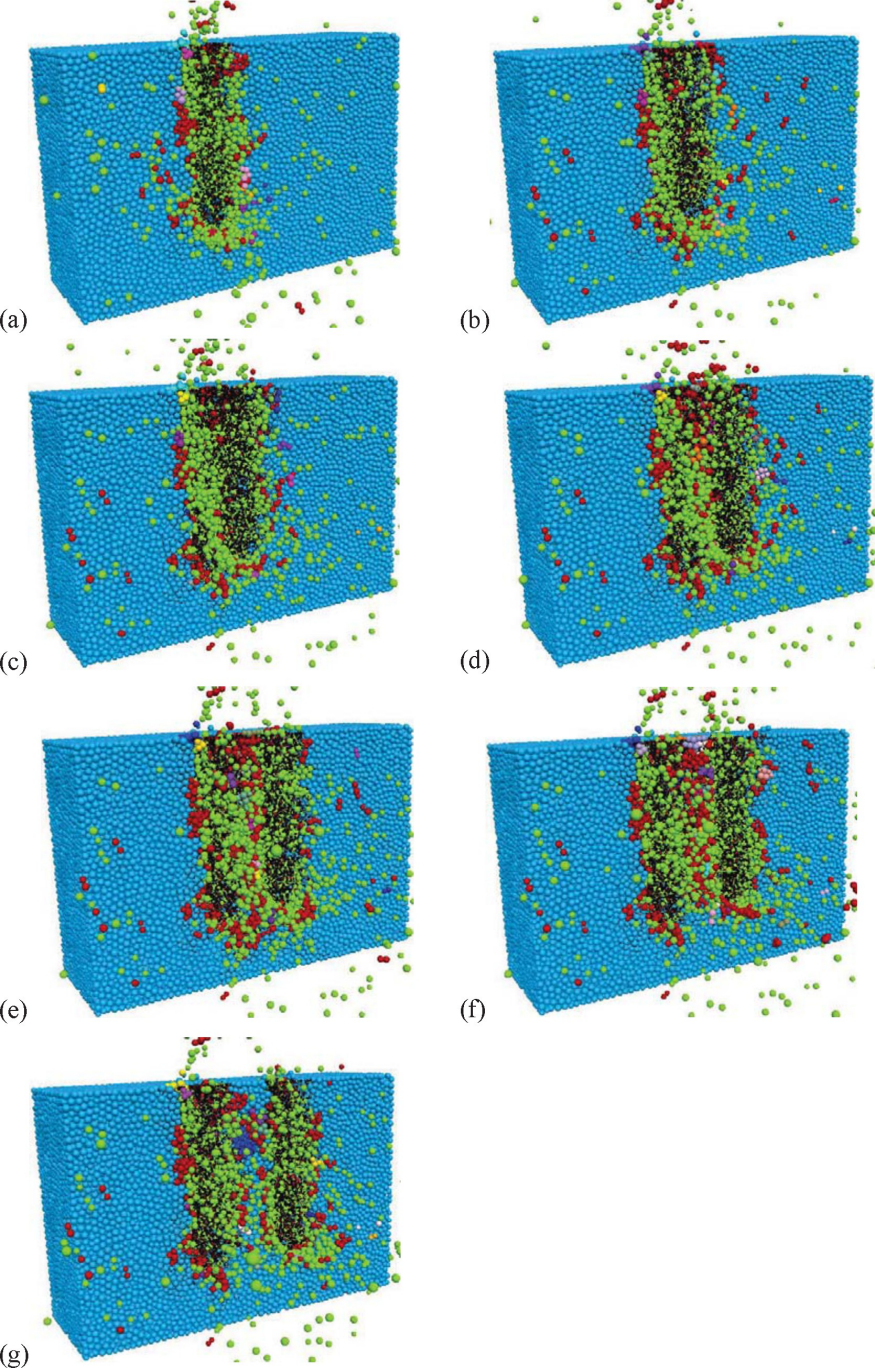
Effect of the cut spacings on rock breakage: (a) cutting process of the first pick, (b)-(g) cutting process of the second pick at s = 6, 10, 16, 20, 25, and 30 mm.

The failure effect of the rock model for different cut spacings is presented in Fig 14. The particles that change color are the parts that fall off, and the red and black cylinders represent the fracture of the bond between the particles due to normal and tangential failure, respectively. Within the simulation, particle color changed when a particle of the rock model lost an inherent contact force, indicating that the particle participated in a crack or a failure. During the initial cutting stage, where the concentrated load acted on the rock specimen, normal cracking occurred. Under complex conditions, three applied forces fragmented the rock specimen during the main cutting stage. Compared with the initial cutting stage, the crack gradually expanded to the left and right sides since the applied area increased as the pick advanced. Additionally, due to the influence of the precedent crack caused by continual cutting, fragmentation in the main cutting stage occurred along the vulnerable crack.

The grain failure number increased with the cut spacing. After the first pick completed the cutting process (Fig 14A), the second pick was offset horizontally to the left by 6 mm, 10 mm, 16 mm, 20 mm, 25 mm, and 30 mm, as shown in Fig 14B–14G. When the cut spacing was too narrow, partially repeated cutting occurred. Moreover, the specific energy was too high, and the rock-breaking effect was relatively poor, as shown in Fig 14B–14G. For the cut spacings of 16 mm, 20 mm, and 25 mm, the cutting grooves produced by two connected adjacent picks effectively broke the rock, as shown in Fig 14D–14F, respectively. When the cut spacing was too wide, a significant ridge appeared between the two cutting grooves, and cracks formed by the adjacent picks did not fully penetrate.

The rock-breaking effect was experimentally analyzed under the condition of constant cutting depth and different cut spacings. The results show that numerical simulations can be used to find an optimum ratio of cut spacing to cutting depth (*s*/*d*) when different hardness rocks are cut by tools. Based on the optimum *s*/*d* ratio, design and optimization of the pick arrangement can be performed.

**Fig 15:**
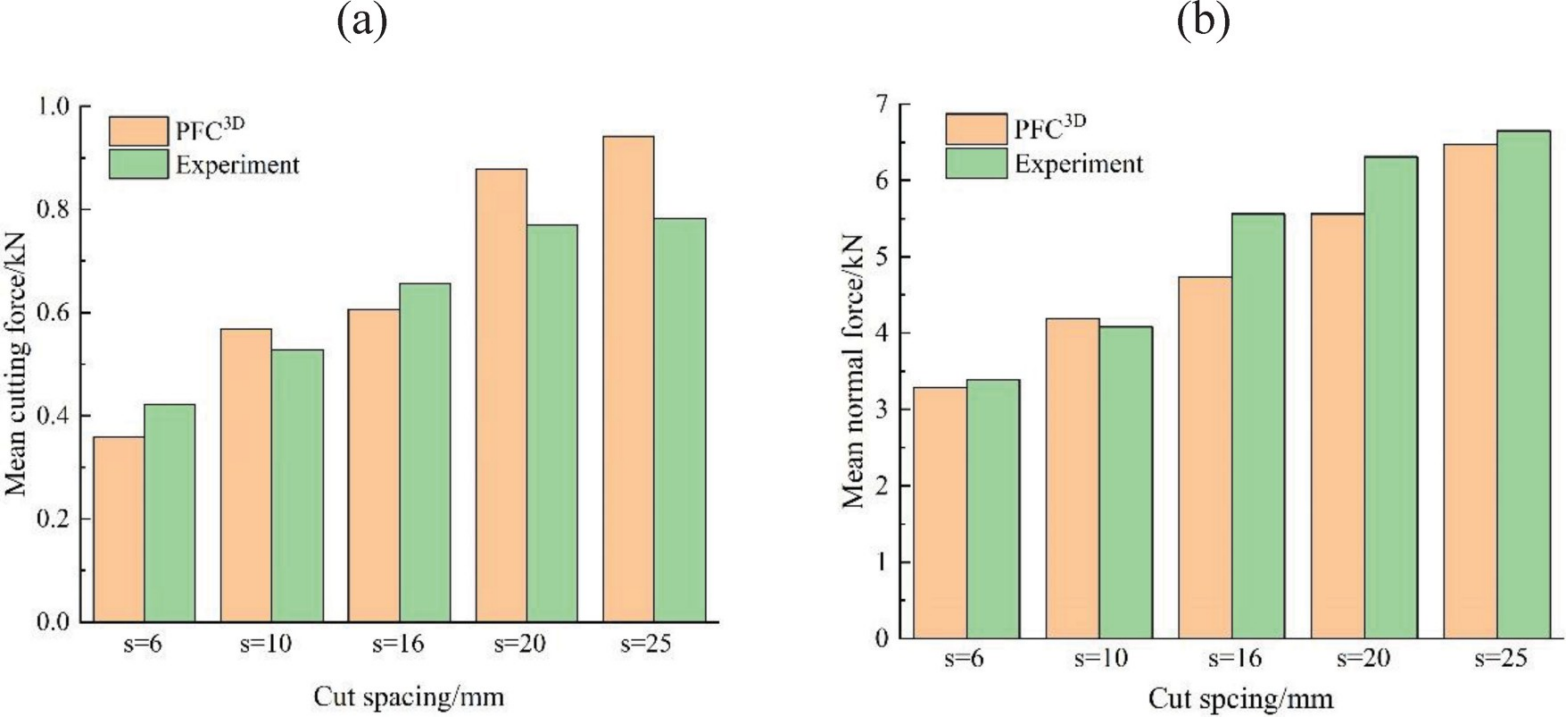
Comparison of numerical and experimental results: (a) mean cutting force, and (b) mean normal force.

To further verify the reliability and accuracy of the particle flow in the discrete element method in the simulation rock cutting process, in terms of the cutting load, the mean cutting force and mean normal force of all groups were compared with the corresponding test results. The parallel bond model between particles can be employed to simulate brittle failure characteristics of the rock. However, it cannot reflect the ratio between the tensile and compression and strength envelope of the rock. The main reason is that the parallel bond model neglects the influence of normal force on the bond strength. Affected by the aforementioned factors, the comparison results showed that the mean cutting force obtained by the numerical simulation method was approximately 8%-20% higher than the corresponding experimental result, and the simulated mean normal force was approximately 3%-15% less than the corresponding experimental result, as shown in Fig 15. Consequently, the pick-rock model built by the PFC^3D^ is reasonable and reliable for predicting loads acting on the pick under varying cutting conditions.

## Conclusions

In this paper, experiments and numerical simulations of rock cutting with conical picks were presented. Based on the obtained results, the following conclusions can be drawn:

The experiment was performed using different combinations of cut spacings and cutting depth. The effects of the *s*/*d* ratio on specific energy, forces, and CI were analyzed. When the rock type and pick were given, an optimal *s*/*d* ratio with a good rock-breaking effect and low specific energy was obtained. Reasonable cutting depth was conducive to improving productivity. At an optimum *s*/*d* ratio, the number of rock chips was maximized, and the amount of dust was minimized. In addition, the specific energy was inversely proportional to the CI.

A pick-rock numerical model was established using the PFC^3D^ software, and the rock cutting process was simulated. The dynamic rock-breaking process was observed from a microscopic perspective, including the generation, extension, and intersection of cracks. Moreover, the forces acting on the picks were accurately predicted. The results of this study are that tensile strength and compressive strength are taken as evaluation indexes in the calibration of microscopic parameters, and the UCS and BTS test conditions of international standards are not implemented. The results have shown that the discrete element method can be used to predict the pick cutting performance, evaluate the rock-breaking effect, and optimize the structural parameters of the cutter head.

The research content of the paper was provided as a guide to optimize the pick arrangement of the cutting head, and the results of numerical simulation are also verified by the experimental data. In the future, PFC^3D^ will be used to further study the numerical simulation of the entire cutting head involved in rock cutting.

## Supporting information

S1 Dataset (DOC)

## References

[1] Jeong H.Y., Cho J.W., Jeon S.W. and Rostami J. Performance assessment of hard rock TBM and rock boreability using punch penetration test. Rock Mech Rock Eng, 2016, 49(4): 1517–1532. DOI: 10.1007/s00603-015-0834-7

[2] Su O., Akcin N.A. Numerical simulation of rock cutting using the discrete element method. Int J Rock Mech Min, 2011, 48(3): 434–442. DOI: 10.1016/j.ijrmms.2010.08.012

[3] Jeong H.Y., Jeon S.K. Characteristic of size distribution of rock chip produced by rock cutting with a pick cutter. Geomech Eng, 2018, 15(3): 811–822. doi: 10.12989/GAE.2018.15.3.811

[4] Bilgin N, Copur H. Dominant rock properties affecting the performance of conical picks and the comparison of some experimental and theoretical results. Int J Rock Mech Min, 2006, 43(5): 139–156.

[5] Evans I. A theory of the cutting force for point-attack picks. Geotech Geolog Eng, 1984, 2(1): 63–71.

[6] Roxborough Ff L Z. Throretical considerations on pick shape in rock and coal cutting, Proceedings of the sixth underground operator’s conference, 1995: 189–193.

[7] Gokan R M. A suggested improvement on Evans’ cutting theory for conical bits. Proceedings of the Fourth symposium on Mine Mechanization Automation, 1997: 57–61.

[8] Goktan R M, Gunes N. A semi-empirical approach to cutting force prediction for point-attack picks. J S Afr I Min Metall, 2005, 105(4): 257–263.

[9] Yilmaz N G, Yurdakul M, Goktan R M. Prediction of radial bit cutting force in high-strength rocks using multiple linear regression analysis. Int J Rock Mech Min, 2007, 44(6): 962–970. DOI: 10.1016/j.ijrmms.2007.02.005

[10] Gao K, Du C, Jiang H, et al. A theoretical model for predicting the Peak Cutting Force of conical picks. Frattura Ed Integrità Strutturale, 2014, 8(27): 43–52.

[11] Li X., Wang S., Ge S., Malekian R., Li Z. A theoretical model for estimating the peak cutting force of conical picks, Exp Mech, 2018, 58: 709–720.

[12] Kang H., Cho J.W., Park J.Y., Jang J.S., Kim J.H., Kim K.W., et al. A new linear cutting machine for assessing the rock-cutting performance of a pick cutter. Int J Rock Mech Min. 2016, 88: 129–136. DOI: 10.1016/j.ijrmms.2016.07.021

[13] Shao W, Li X, Sun Y, et al. An experimental study of temperature at the tip of point-attack pick during rock cutting process. Int J Rock Mech Min, 2018, 107: 39–47.

[14] Wang X, Su O, Wang Q F, et al. Effect of cutting depth and line spacing on the cuttability behavior of sandstones by conical picks. Arab J Geosci, 2017, 10(23): 525.

[15] Wang Z, Zhou J, Lei Y, Du W. Bearing fault diagnosis method based on adaptive maximum cyclostationarity blind deconvolution. Mech Syst Signal Pr, 2022, 162: 108018.

[16] Wang Z, Yang N, Li N. A new fault diagnosis method based on adaptive spectrum mode extraction[J]. Structural Health Monitoring, 2021. doi: 10.1177/1475921720986945

[17] Wang Z, Zhao W, Du W, Li N, Wang J. Data-driven fault diagnosis method based on the conversion of erosion operation signals into images and Convolutional Neural Network. Process Saf Environ Prot. 2021, 149: 591–601.

[18] Dai X., Huang Z, Shi H, Wu X, Xiong C. Cutting force as an index to identify the ductile-brittle failure modes in rock cutting. Int J Rock Mech Min, 2021, 146, 104834. R. DOI: 10.1016/j.ijrmms.2021.104834

[19] Comakli C. Balci H. Copur D. Tumac. Experimental studies using a new portable linear rock cutting machine and verification for disc cutters. Tunn Undergr Space Technol, 2021, 108, 103702.

[20] Su O, Akcin NA. Modeling of unrelieved rock cutting test by using PFC3D. In: Proceedings of the first international FLAC/DEM symposium, Minneapolis, 2008, 165–172.

[21] Su O. Numerical simulation of rock cutting using the discrete element method. Int J Rock Mech Min. 2011, 48: 434–442.

[22] Moon T, Oh J. A Study of Optimal Rock-Cutting Conditions for Hard Rock TBM Using the Discrete Element Method. Rock Mech Rock Eng. 2012, 45:837–849. DOI: 10.1007/s00603-011-0180-3

[23] Van Wyk G, Els D.N.J, Akdogan G., Bradshaw S.W., Sacks N. Discrete element simulation of tribological interactions in rock cutting. Int J Rock Mech Min, 2014, 65: 8–19.

[24] Mc Feat-Smith, I, R J Fowell. Correlation of rock properties and the cutting performance of tunnelling machines. Proceedings of a Conference on Rock Engineering, 1977: 581–602.

[25] Rostami J, Ozdemir L. Modeling for design and performance analysis of mechanical excavators. Proceedings of the Conference on Mechanical Excavation’s Future Role in Mining, 1996.

[26] Tuncdemir H, Bilgin N, Copur H, et al. Control of rock cutting efficiency by muck size. Int J Rock Mech Min, 2008, 45(2): 278–288.

[27] Bakar M A Z, Gertsch L S, Rostami J. Evaluation of Fragments from Disc Cutting of Dry and Saturated Sandstone. Rock Mech Rock Eng, 2014, 47(5): 1891–1903.

[28] Bakar M Z A, Gertsch L S. Evaluation of saturation effects on drag pick cutting of a brittle sandstone from full scale linear cutting tests. Tunn Undergr Sp Tech, 2013, 34(1): 124–134. DOI: 10.1016/j.tust.2012.11.009

[29] Cundall P.A., Hart D.H. Numerical modelling of discontinue. Eng Comput, 1992, 9: 101–113.

[30] Itasca Consulting Group. Particle flow code in 3 dimensions manual, version3.1. 2006, Minneapolis, Itasca.

[31] Potyondy D O, Cundall P A. A bonded-particle model for rock. Int J Rock Mech Min, 2004, 41(8): 1329–1364. doi: 10.1016/j.ijrmms.2004.09.011

