# Supplementary material for: A study on pick cutting properties with full-scale rotary cutting experiments and numerical simulations
Source: PLoS One. 2022 Apr 14;17(4):e0266872. doi: 10.1371/journal.pone.0266872 (PMC9009718; doi:10.1371/journal.pone.0266872)
Supplement: S1 Dataset — (DOC) [file pone.0266872.s001.doc]

**The underlying data supporting the results of the study are listed in follows:**

**Figure 7:**

s/d:

5

8

10

12.5

1.5

4

5

6.25

1

1.67

2.67

3.33

4.17

0.75

1.25

2

2.5

3.13

Specific Energy:

40.9

55.8

64.94

60.69

58.19

33.48

37.39

39.14

40.37

35.68

29.75

25.71

27.77

35.26

30.31

25.12

23.37

22.16 (MJ/m^3^)

**Figure 8:**

Cut spacing: 6 10 16 20 25 (mm)

The mean cutting force data when d=2 mm: 0.351 0.447 0.577 0.565 0.663 (kN)

The mean cutting force data when d=4 mm: 0.422 0.527 0.656 0.769 0.782 (kN)

The mean cutting force data when d=6 mm: 0.494 0.775 1.048 1.204 1.261 (kN)

The mean cutting force data when d=8 mm: 0.847 1.257 1.446 1.587 1.696 (kN)

**Figure 9:**

Cut spacing: 6 10 16 20 25 (mm)

The mean normal force data when d=2 mm: 2.8 3.9 4.28 4.18 5.12 (kN)

The mean normal force data when d=4 mm: 3.39 4.08 5.56 6.31 6.65 (kN)

The mean normal force data when d=6 mm: 4.11 6.4 8.52 9 10.09 (kN)

The mean normal force data when d=8 mm: 6.409 9.129 10.787 11.912 12.843 (kN)

**Figure 11:**

CI:

138.06

228.6

189.48

174.64

181.64

161.13

182.28

258.06

226.01

201.49

167.56

185.35

259.93

289.9

353.55

175.63

196.24

299.43

364.58

383.76

SE:

67.64

40.9

55.8

64.94

60.69

63.19

43.96

33.48

37.39

39.14

40.37

35.68

29.75

25.71

27.77

35.26

30.31

25.12

23.37

22.16

**Figure 15:**

| Cut spacing/mm | | 6 | 10 | 16 | 20 | 25 |
| --- | --- | --- | --- | --- | --- | --- |
| Experiment | Mean cutting force/kN | 0.422 | 0.527 | 0.656 | 0.769 | 0.782 |
|  | Mean normal force/kN | 3.39 | 4.08 | 5.56 | 6.31 | 6.65 |
| PFC^3D^ | Mean cutting force/kN | 0.359 | 0.569 | 0.606 | 0.878 | 0.942 |
|  | Mean normal force/kN | 3.289 | 4.189 | 4.735 | 5.563 | 6.478 |
